# Determination of Benzo[a]pyrene in Traditional, Industrial and Semi- industrial Breads Using a Modified QuEChERS Extraction, Dispersive SPE and GC-MS and Estimation of its Dietary Intake

**Published:** 2016

**Authors:** Samira Eslamizad, Farzad Kobarfard, Katayon Javidnia, Ramezan Sadeghi, Mitra Bayat, Sara Shahanipour, Najmeh Khalighian, Hassan Yazdanpanah

**Affiliations:** a *Food Safety Research Center, Shahid Beheshti University of Medical Sciences, Tehran, Iran. *; b *Department of Medicinal Chemistry, School of Pharmacy, Shahid Beheshti University of Medical Sciences, Tehran, Iran. *; c *Phytochemistry Research Center, Shahid Beheshti University of Medical Sciences, Tehran, Iran. *; d *Medicinal and Natural Products Chemistry Research Centre, Shiraz University of Medical Sciences, Shiraz, Iran. *; e *Department of Environmental Health Engineering, School of Health, Shahrekord University of Medical Sciences, Shahrekord, Iran. *; f *Department of Toxicology and Pharmacology, School of Pharmacy, Shahid Beheshti University of Medical Sciences, Tehran, Iran.*

**Keywords:** Benzo[a]pyrene, Traditional bread, Industrial bread, Semi-industrial bread, Sangak bread, QuEChERS (quick, easy, cheap, effective, rugged and safe, GC-MS( Gas Chromatography- Mass Spectroscopy), Tehran, Shiraz, Iran

## Abstract

A fast and simple modified QuEChERS extraction method was developed for determination of Benzo[a]pyrene (BaP) in 137 traditional (Sangak), semi-industrial (Sangak) and industrial bread samples using spiked calibration curves by GC/MS. Sample preparation includes extraction of BaP into acetone followed by cleanup with dispersive solid phase extraction. The limit of detection and limit of quantification were 0.3 ng/g and 0.5 ng/g, respectively. The values for recoveries and RSD were calculated as 110.5-119.85% and <11.6% respectively. Average concentration of BaP in Sangak bread samples of Shiraz and Tehran was 0.59 and 0.60 ng/g, respectively. 35.5% of samples of breads collected in Tehran were contaminated with BaP at the amount higher than maximum levels regulated in processed cereal-based foods and baby foods by European Commission (1 ng/g). Seventeen percent of samples of breads collected in Shiraz were contaminated with BaP which 13 % of total samples were >1 ng/g. BaP content in all industrial samples was lower than LOQ.

Assuming the consumption of bread in Tehran and Shiraz is limited to these kinds of breads, the daily intake of BaP in Tehran and Shiraz population through bread consumption was estimated to be 170.6 and 168.7 ng/day, respectively. This is the first report concerning contamination of bread samples with BaP in Iran.

## Introduction

Benzo[a]pyrene (BaP) is a polycyclic aromatic hydrocarbon (PAH) that is a byproduct of incomplete combustion or pyrolysis (burning) of organic substance such as cigarettes, gasoline, wood, waste or food, during industrial processes and other human activities (1, 2). BaP is also found in ambient (outdoor) air, indoor air, and in some water sources (3). International Agency for Research on Cancer (IARC) reported that BaP is carcinogenic to humans (Group 1) (4).

Toxicological studies on individual PAHs in animals, chiefly on BaP, have revealed various toxicological effects, such as hematological effects, immunotoxicity, and reproductive and developmental toxicity (1). BaP is metabolized in humans and animals to form a number of metabolites that may elicit toxicity. BaP and BaP metabolites can bind to DNA forming a structure called BaP-DNA adducts. The formation of BaP-DNA adducts can interfere with or alter DNA replication (formation of DNA copies during cell division), and may be related with an increased risk of several forms of cancer (2). SCF (Scientific Committee on Food) has concluded that, according to toxicological information, BaP is a suitable marker of the occurrence of the carcinogenic PAHs in food and their health effects (5). 

On the basis of the studies on PAHs exposure, in the non-tobacco-smoking and non-occupationally exposed population, the main source of human exposure to PAHs is food, and cereals constitute one of the major contributing sources (6-11).

In food, PAHs are produced during thermal food processing or certain home cooking practices such as smoking, barbecuing, roasting, grilling, drying, baking and frying (7).

Bread is a very important food in human nourishment. It is a good source of energy, contains vitamins, proteins, lipids and minerals, which are essential in human diet (12). Kazerouni (13) reported that bread and other cereal products (29%) followed by grilled/barbecued meat (21%) contributed most to the total daily intake of BaP in USA. Also in China it was declared that three main sources of dietary PAHs were vegetables, wheat flour and fruits, the sum of which contributes 75.95% of PAHs in dietary food (14).

In Iran, there are three types of bread available in the market including traditional, industrial and semi-industrial bread. Bread’s contamination by PAHs can be dependent on both the contamination of raw materials, primarily flour, and the baking process. An important issue is also the temperature of thermal treatment and its influence on bread’s contamination level (7).

BaP is the only PAHs with enough toxicological evidence to allow the setting of a guideline (15)**.** According to existing regulations by European Commission (16), the content of BaP in some foods and baby foods should not exceed 1 ppb (17). In Iran, National Standard Organization is setting maximum limit for PAH in foods. 

In Iran, a few studies have been done concerning determination of BaP in different matrices such as: water (sea water, drinking water, surface water and urban run-off) (18-21), atmosphere (22), soil (23, 24), type of Sediments (25-27) and Plants (28). But, there is no information regarding contamination of foods including bread with Bap in Iran. Therefore, the aim of this study was to evaluate contamination of Iranian bread samples (traditional, industrial and semi-industrial) with BaP using a QuEChERS method and GC/MS analysis. The daily intake of BaP through bread consumption was also calculated. 

Common preparation techniques for assessing PAH contamination in different food matrices include solid-liquid extraction, liquid-liquid extraction, soxhlet extraction, sonication assisted extraction, microwave-assisted extraction (29-33).

Nevertheless, traditional methods are expensive, solvent intense and time-consuming and require advanced analytical equipment (32). To overcome these challenges, for the first time, a QuEChERS (quick, easy, cheap, effective, rugged and safe) extraction and clean up method for the analysis of BaP in bread using GC/MS was developed (34) Several QuEChERS methods have been developed for the analysis of PAHs in seafood such as shrimp, and fish (32).

## Experimental

*Sample collection*

Twenty nine traditional and forty seven semi-industrial bread Samples were collected between July 2012 to February 2014 from Sangak bakeries located in Shiraz city (located in west south of Iran). Between January 2014 to February 2014, 31 traditional bread Samples and 30 industrial breads were collected from Sangak bakeries and retail stores located in Tehran City, respectively. All bread samples were baked from wheat flour. After collection, all samples were covered with aluminum foil in order to prevent photodegradation and transported to the lab. Each sample was separately cut into small pieces and blended. After mixing, the samples were stored in amber glass bottles with Teflon-lined caps at −20 ^◦^C until analysis. 

*Chemicals*

BaP and anthracene-d10 as internal standard (ISTD) were purchased from Sigma Aldrich (St. Louis, Mo., USA), HPLC grade solvents including acetonitrile, acetone, ethyl acetate, methanol, toluene and isooctane were purchased from Merck (Darmstadt, Germany). Sodium chloride and anhydrous MgSO_4_ were obtained from Chem Lab NV, Belgium and Primary Secondary Amine (PSA) SPE Bulk Sorbent was purchased from Varian, Italy. Ultrapure water was prepared using a water purification system (Econolab. Oklahama, USA). 

*Preparation of standards*

BaP and anthracene-d10 stock solutions were prepared at 1 mg/mL concentration in toluene. Intermediate standard solutions of BaP included concentrations of 10,000, 1000 and 100 ng/mL and intermediate standard solutions for anthracene-d10 included 10,000 and 1000 ng/mL were made in acetone. Bread samples was Spiked with BaP working standards in acetone at: 500, 250, 125, 50, 25, 12.5, and 5 ng/mL. 200 µL of each working standard solution was added to 5 g of blank bread samples. Fifty μL of anthracene-d10 solution in acetone (1000 ng/mL) was added to the all spiked bread samples.

To avoid light exposure, all standard solutions were made in amber color volumetric flask and stored at 4 °C whenever not in use. The samples so obtained were treated as described in sample preparation section.

*QuEChERS Sample preparation*

 The QuEChERS method developed for the analysis of pesticide by Anastassiades *et al*. (35), was used with some modifications for the analysis of BaP in bread. The extraction procedure is as follows: 5 g sample was weighed into a 50 mL “PAH free” centrifuge tube, and 50 μL of 1000 ng/mL of the ISTD solution, 5 mL deionized water and then 10 mL acetone were added. The tube was shaken by vortex shaker for 60 s, then 6 g MgSO_4_ and 1.5 g NaCl were added to the tube. The mixture was immediately shaken by hand followed by vortex shaker for 30 s. The mixture was then centrifuged at 4000 RPM for 5 min. The whole acetone extract was transferred to a dispersive SPE cleanup tube contained 400 mg PSA and 1200 mg MgSO4 and shaken by vortex shaker for 30 s. The dispersive SPE tube was centrifuged at 4000 RPM for 5 min and then 6 mL of the extract was transferred into a 10 mL amber vial and dried under gentle flow of nitrogen gas at ambient temperature. The dried extract was re-dissolved in 100 µL of acetone by vortex shaking for 30 s and followed by sonication for 60 s. Finally, the whole extract was transferred to an amber vial.

*Gas Chromatography—Mass Spectrometry Condition*

BaP analysis was performed using an Agilent 7000-Triple-Quad mass spectrometer coupled with 7890A gas chromatography. Separation of BaP and anthracene-d10 was conducted using a 5% phenyl-methylsilicone (HB-5MS) bonded-phase fused-silica capillary column (Hewlett-Packard, 30 m *× *0.25 mm i.d., film thickness 0.25 *μ*m). The carrier gas was helium. The injection port was adjusted in splitless mode and the injection volume was 2 *μ*L. The total run time was 15.2 minutes and the oven temperature program was 80 ºC for 1.5 min, raised to 290 ºC at a rate of 50 ºC/min and maintained at this temperature for 10 min. The MS transfer line and ion source temperatures were adjusted at 290 ºC and 230 ºC, respectively. The mass spectra were collected at 70 eV by electron impact. Detection of B*a*P and ISTD was performed using SIM mode. Quantifier ions and qualifier ions are show in table 1. Determination was carried out based on the ratio between the peak area of the B*a*P to that of the ISTD. 

**Table 1:** Quantifier ions and qualifier ions used in the selected ion-monitoring of BaP and Anthracene-d10 by gas chromatography-mass spectrometry

**Compound**	**Quantifier ion**	**Qualifier ion**
BaP	252	253, 250, 126
Anthracene-d10	188	189, 187, 160

## Results

*Evaluation of performance characteristic of the method*

*Method validation*

The linearity of the method for the analysis of Bap was evaluated by building the spiked calibration curves over the BaP concentration range of 0.5–20 ng/g. The correlation coefficient was 0.997. 

The data of performance characteristic of the method are shown in table 2. Average recoveries and repeatabilities at three spiked levels (1, 3, 16 ng/g) were in the range of 97-120% and 1-14.5, respectively, that are in accordance with the criteria set by European Commission (36). Measurement uncertainty of the method was 0.19 ng/g. The value of Limit of detection (LOD) was 0.3 ng/g. The obtained HorRAT values ranged from 0.4 to 0.5 which is lower than the criteria set by European Commission (36).

**Table 2:** Recovery, RSD, LOD and LOQ of the method for analysis of BaP in bread samples.

**Matrix**	**LOD (ng/g)**	** LOQ (ng/g)**	**Recovery** **(%)**	**RSD**_R_ ** (%)** **(n= 3)**	**Measurement ** **uncertainty (ng/g)**
Bread	0.3	0.5	97–120%	4.40	0.19

*Quality control samples (QC)*

Eight quality control (QC) bread samples spiked at the level of 8 ng/g were carried out in each working round. Average recovery and RSD (%) of QC samples are shown in table 3. Average recovery and RSD (%) of QC samples were in accordance with the criteria set by European Commission (36).

**Table 3:** Average recovery and RSD (%) of QC samples used for analysis of BaP

**Type of sample**	**Average recovery ± RSD (%)**
Sangak bread of Shiraz (n= 4)	104.23 ± 6.02
Sangak bread of Tehran (n= 2)	98.30 ± 18.11
Industrial bread of Tehran (n= 2)	101.34 ± 15.57

*Determination of BaP in bread samples*

The results of determination of BaP in bread samples are presented in tables 4 and 5. The results indicate that 35.5% of the samples collected in Tehran, were contaminated with BaP and their contamination was higher than maximum level regulated for processed cereal-based foods and baby foods by European Commission (1 ng/g) (16). Seventeen percent of the samples collected in Shiraz were contaminated with BaP which 13% of the samples contained BaP higher than 1 ng/g (16). BaP content in all industrial samples was lower than LOQ. 

As it appears in table 5, average concentration of BaP in all bread samples collected in Shiraz and Tehran was less than the permissible limit of European Commission regulatory control value for B*a*P (1 *μ*g/kg of wet weight) in processed cereal-based foods and baby foods for infants and young children (16). BaP content in all industrial bread samples was lower than the permissible limit of European Commission regulatory control value for B*a*P (16). 

**Figure 1:**
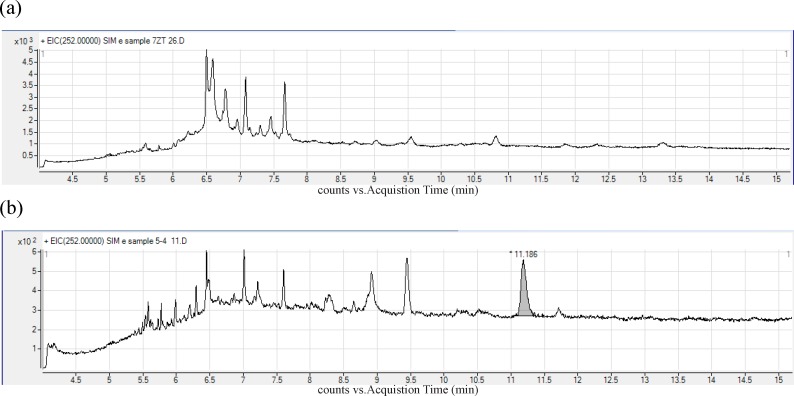
GC-MS chromatogram of BaP in (a) Blank bread sample (b) real contaminated traditional sample

Figure 1 show the chromatograms obtained for (a) Blank bread sample (b) real contaminated traditional sample.

**Table 4:** Occurrence of BaP in Iranian bread samples

**Location**	**Sample** **matrix**	**Numbers of samples**	**Numbers of samples in the range ng/g**		
			**<0.5**	**0.5 – 1**	**> 1**
Shiraz	Semi-industrial Sangak bread	47	39	2	6
	Traditional Sangak bread	29	24	1	4
Tehran	Traditional Sangak bread	31	20	0	11
	Industrial bread	30	30	0	0

**Table 5:** Contamination of Iranian bread samples with BaP (ng/g)*.

**Location**	**Bread type**	**Range**	**Mean**	**Median**	**90th** **percentile**	**97.5th** **percentile**
Shiraz	Semi-industrial Sangak	LOQ-2.46	0.50	0.25	1.35	2.44
	Traditional Sangak	LOQ-7.73	0.72	0.25	1.31	4.92
Tehran	Traditional Sangak	LOQ-3.19	0.93	0.25	2.33	2.84
	Industrial	LOQ-0.25	0.25	0.25	0.25	0.25
Tehran and Shiraz	All samples	LOQ-7.73	0.59	0.25	1.90	2.68

* Mean, Mean, 90th percentile and 97.5th percentile of all samples: Data below LOQ (0.5 ng/g) have been assumed to be 0.25 ng/g.

*Dietary exposure*

To estimate the exposures to BaP associated with the ingestion of traditional, semi-industrial and industrial bread samples, it is essential to determine the dietary intake by the Iranian population.

In the Iranian national nutrition survey, there is no information about the amount of consumption of different Iranian breads. Because of the absence of this information, the estimation of dietary intake for the general population was derived from Iranian total consumption of all types of breads. The data are presented in table 6.

**Table 6:** Dietary exposure to BaP (ng/kg bw/day) through bread consumption in population of Shiraz and Tehran*.

**Location**	**Bread type**	**Mean**	**90th percentile**	**97.5th percentile**	**Max**
Shiraz	Semi-industrial Sangak	2.04	5.51	9.96	10.05
	Traditional Sangak	2.94	5.35	20.10	31.58
Tehran	Traditional Sangak	3.80	9.51	11.60	13.03
	Industrial	1.02	1.02	1.02	1.02
Tehran and Shiraz	All bread	2.42	7.72	10.96	31.58

* Mean, 90th percentile, 97.5th percentile and max of all samples: Data below LOQ (0.5 ng/g) have been assumed to be 0.25 ng/g.

The mean bread consumption for the total population, based on national nutrition survey (37) data, was estimated to be 286 grams per person per day. In this study, average concentration of BaP was calculated in all samples and LOQ/2 was assumed for the concentrations under LOQ. The general Population’s dietary exposure to BaP in Shiraz only through sangak bread assuming a person consumed 50% of traditional sangak bread and 50% semi-industrial sangak bread, was estimated to be 168.7 ng/day. The general population’s dietary exposure to BaP in Tehran only through sangak bread assuming a person consumed 100% of traditional sangak bread was estimated to be 266.4 ng/day. Dietary exposure to BaP in population of Tehran merely through industrial bread, assuming a person consumed 100% of industrial bread, was estimated to be 71.5 ng/day. 

The daily intake of BaP in Tehran populations through traditional and industrial bread consumption, assuming a person consumed 50% of traditional sangak bread and 50% industrial sangak bread was estimated to be 170.6 ng/day.

These results are not comparable to those reported by the EFSA (European Food Safety Authority) for BaP (67 ng/day) in Cereals and cereal products for EU countries (38).

## Discussion

*Occurrence of BaP in bread*

There are only few published papers regarding PAH contamination in bread. 17.5% of the total samples analyzed in the present study were contaminated with BaP. Bread’s contamination by PAHs could be due to both the contamination of bakery raw materials such as water and primarily flour, and the baking process. An important issue is also the temperature of thermal treatment and its influence on bread’s contamination level.

There are some reports regarding PAHs determination in water (18, 39 and 40). Sadeghi *et al*. (40) and Karyab *et al*. (18) have claimed that the mean content of BaP in drinking water of Tehran was lower than 0.7 µg/L [the guideline value for bap in drinking water corresponding to an excess lifetime cancer risk of 10^-5^ proposed by World Health Organization (WHO)] as well as that of Iranian National Drinking Water Standards for all of the samples. 

Jing *et al*. (14) investigated the contribution of each food category (fish, chicken, pork, beef, mutton, vegetables, fruits, milk, eggs, rice, flour and edible oil) to the total PAH levels in raw food and cooked dietary food. Their results showed that contribution of wheat flour category to the total PAH levels in raw food and cooked food was 44 % and 30 %, respectively. Therefore part of the contamination that was observed in Iranian bread samples maybe due to wheat flour contamination.

Tawfic Ahmed *et al*. (41) reported that type of fuel, type of baking thermal treatment (direct or indirect) and temperature of baking affects on content of PAHs in bread. They showed that the BaP levels in bread samples were 20.6, 14.7, 45.1 and 4.4 µg/kg when heavy oil (mazot), light oil (solar), solid waste and electricity were used respectively as heating source for baking process.

Their results showed that fossil fuels could increase the levels of PAHs in bread samples. Since in this study natural gas had been used as fuel for baking traditional sangak breads, part of contamination could have arisen because of the use of gas as the fuel.

Rey-Salgueiro *et al.* (42) reported the levels of BaP in sandwich bread with several treatment conditions, direct toasting (flame-toasting, coal-grilling or gas oven-toasting) or indirect toasting (electric oven-toasting). In indirect toasting, BaP was not detected in Electric oven at 200 °C and Toaster at 250–270 °C. BaP concentration detected in Muffle at 300 °C and Muffle at 500 °C had an average of 0.5 and 0.8 µg kg^−^^1^, respectively. 

In this study traditional bread have been baked by direct heating in the temperature range of 242-352 °C. Semi-industrial bread have been baked by indirect heating at temperatures between 160 and 470 °C in different times but industrial breads have been baked by hot oil via indirect temperature on hot plate at maximum temperature of 220 °C. Since, PAHs can be formed at temperatures between 300 and 600 °C, it is possible that part of bread contamination with BaP has been arisen due to the high temprature during baking process.

*Dietary exposure*

A summary of data on dietary intakes of PAHs in various countries including Iran is presented in Table 7. 

**Table 7:** Dietary intakes of PAHs: Results of a number of international Surveys

**Survey country**	**Year of publication**	**No. of single** **PAHs analyzed**	**Intake** **(µg/person/day)**	**Increase risk of cancer**	**Reference**
United Kingdom	1983	11	3.70	74/10^6^	***(43)***
The Netherlands	1990	17	5–17	100-340/10^6^	***(44)***
Italy	1995	9	3	60/10^6^	**(9)**
Greece^a^	1998	16	1.6–4.5	32-90/10^6^	**(45)**
Spain (Catalonia)	2003	16	6.33^b^–8.42^c^	126.5-168.5/10^6^	**(8)**
Spain (Catalonia)	*2010*	*16*	*6.72*	134.5/10^6^	**(46)**
*Poland* d	*2013*	*19*	*1.54*	31/10^6^	**(7)**
*USA*	*2001*	*BaP*	*0.04-0.06*	1/10^6^	**(13)**
*Efsa study (16 European countries)*	*2008*	*8*	*0.393* ^f^	7.86/10^6^	**(38)**
*Iran* e	*2014*	*BaP*	*0.17*	3.5/10^6^	***This study***

a Only dietary intake via vegetables was estimated.

b Intake for seniors.

c Intake for male adults

d bakery chain, its raw materials and final products

e Only dietary intake via bread was estimated

f Only dietary intake via Cereals and cereal products was estimated

On the basis of animal carcinogenicity data, an acceptable daily intake of BaP has been computed as the quantity that would be associated with a 1/10^6^ increase in risk of cancer for an adult of 70 kg*. *This risk increase level corresponds to a dietary intake level of approximately 0.05 µg/day (8, 45). The estimated total daily intake of BaP by Iranian people via bread can be associated with only 3.5/1000000 increased risk for cancer development. 

On the basis of the results of the present study and with the intention of decreasing the intake of carcinogenic substances, a recommendation concerning the decreasing baking temperature (less than about 220 °C) and not using open fire for baking are recommended. 

As human exposure to environmental carcinogens such as PAHs arises predominantly from dietary sources, further studies will be necessary to establish the trend for PAH concentrations in foodstuffs as well as the trend for their dietary intake.

## Conclusions

To the best of our knowledge, there are no published QuEChERS extraction and clean up method to determine BaP in bread samples. The proposed method offers an efficient, cost effective, less time consuming and easy sample preparation procedure for the determination of BaP in bread. The extraction method is relatively simple and cheap compared to other extraction methods. The use of spiked calibration curves for constructing the calibration curve substantially reduced adverse matrix-related effects.

The results reveal that BaP concentrations in traditional bread samples are higher than maximum levels set for processed cereal-based foods and baby foods (16).

BaP content in industrial bread samples was under LOQ. Although BaP concentrations in the traditional and semi-industrial samples are less than maximum levels regulated in processed cereal-based foods and baby foods (16) but average dietary exposure in Shiraz and Tehran only through bread samples was higher than mean dietary consumers across European countries (0.95 ng/kg b.w. per day) through cereals and cereal products. Therefore, further studies are suggested for monitoring of BaP and other PAHs in different food products and estimating of average dietary exposure through all food products. 
